# "I tell you, getting data for this is hell"–Exploring the use of evidence for noncommunicable disease policies in Ghana

**DOI:** 10.1371/journal.pgph.0002308

**Published:** 2023-08-24

**Authors:** Mark Fordjour Owusu, Joseph Adu, Benjamin Ansah Dortey

**Affiliations:** 1 School of Health Sciences, University of Canterbury, Christchurch, New Zealand; 2 Department of Health and Rehabilitation, Western University, London, Ontario, Canada; 3 Trauma and Specialist Hospital, Winneba, Ghana; University of Washington, UNITED STATES

## Abstract

After several years of over concentration on communicable diseases, Ghana has finally made notable strides in the prevention of NCDs by introducing key policies and programmes. Evident shows that there is limited NCD-related data on mortality and risk factors to inform NCD policy, planning, and implementation in Ghana. We explored the evidence base for noncommunicable disease policies in Ghana. A qualitative approach was adopted using key informant interviews and documents as data sources. An adaptation of the framework method for analysing qualitative data by Gale and colleagues’ (2013) was used to analyse data. Our findings show that effort has been made in terms of institutions and systems to provide evidence for the policy process with the creation of the Centre for Health Information Management and the District Health Information Management System. Although there is overreliance on routine facility data, policies have also been framed using surveys, burden of disease estimates, monitoring reports, and systematic reviews. There is little emphasis on content analysis, key informant interviews, case studies, and implementation science techniques in the policy process of Ghana. Inadequate and poor data quality are key challenges that confront policymakers. Ghana has improved its information infrastructure but access to quality noncommunicable disease data remains a daunting challenge. A broader framework for the integration of different sources of data such as verbal autopsies and natural experiments is needed while strengthening existing systems. This, however, requires greater investments in personnel and logistics at national and district levels.

## Introduction and background

In Africa, access to evidence for health policies remains a daunting challenge [1, 2]. Following the 2011 UN Global Summit, the use of evidence has been heralded as key to the development of non-communicable disease (NCD) policies. Prior to the Summit, the World Health Organisation (WHO) highlighted the role of evidence to informing and shaping NCD prevention and control policies and strategies which became a key aspect of the overarching principles of the Global Action Plan for the Prevention and Control of Noncommunicable Diseases 2013–2020. However, evidence shows that a significant proportion of data to inform NCD policy has been largely derived from High-Income Countries (HICs) [3]. Nyaaba et al [4] in their synthesis of Africa’s progress toward implementing the NCD Global Action Plan found that only 3.7% of countries in Africa had effective systems for generating cause-specific mortality data in 2015 although this is crucial to NCD policy development and implementation. This is partly attributed to a traditional health policy and systems focus on preventing and treating infectious diseases in Africa to the neglect of NCDs despite an increasing double-burden of diseases [5–7].

After years of grappling with communicable diseases, Ghana, a West African country, has finally made notable strides in the prevention of NCDs by introducing key policies and programmes following the rising incidence of these conditions in the country [8–10]. The current NCD policy, developed in 2022, aims to consolidate the gains made after the previous policy which was developed in 2012. The policy attempts to align Ghana’s NCD goals and strategies with global frameworks including the Sustainable Development Goals (SDGs). However, the sources of evidence for the development of the overarching NCD policy and other policies remain unclear. This is stated in the policy document, with the framers admitting that “very limited population based NCD data exist” in Ghana [10 p.9]. A number of studies have been conducted on NCD policies in Ghana [11–13]. However, the Non-communicable Disease Progress Monitor 2022 [14] shows that there is limited NCD-related data on mortality and risk factors to inform NCD policy, planning and implementation in Ghana. This is crucial evidence needed for effective NCD policy, planning and implementation to achieve population level benefits. Thus, more studies are needed on the nature and sources of evidence for these policies. This is important as researchers agree that health programmes and policies likely to have the desired outcomes are those that are based on robust evidence [3, 15]. There is, therefore, a strong case for investigating the NCD evidence scenario and the likely consequences of this on the policy process. This paper, therefore, aims to explore the evidential underpinnings of NCD policies in Ghana.

### Conceptual framework

**Fig 1:**
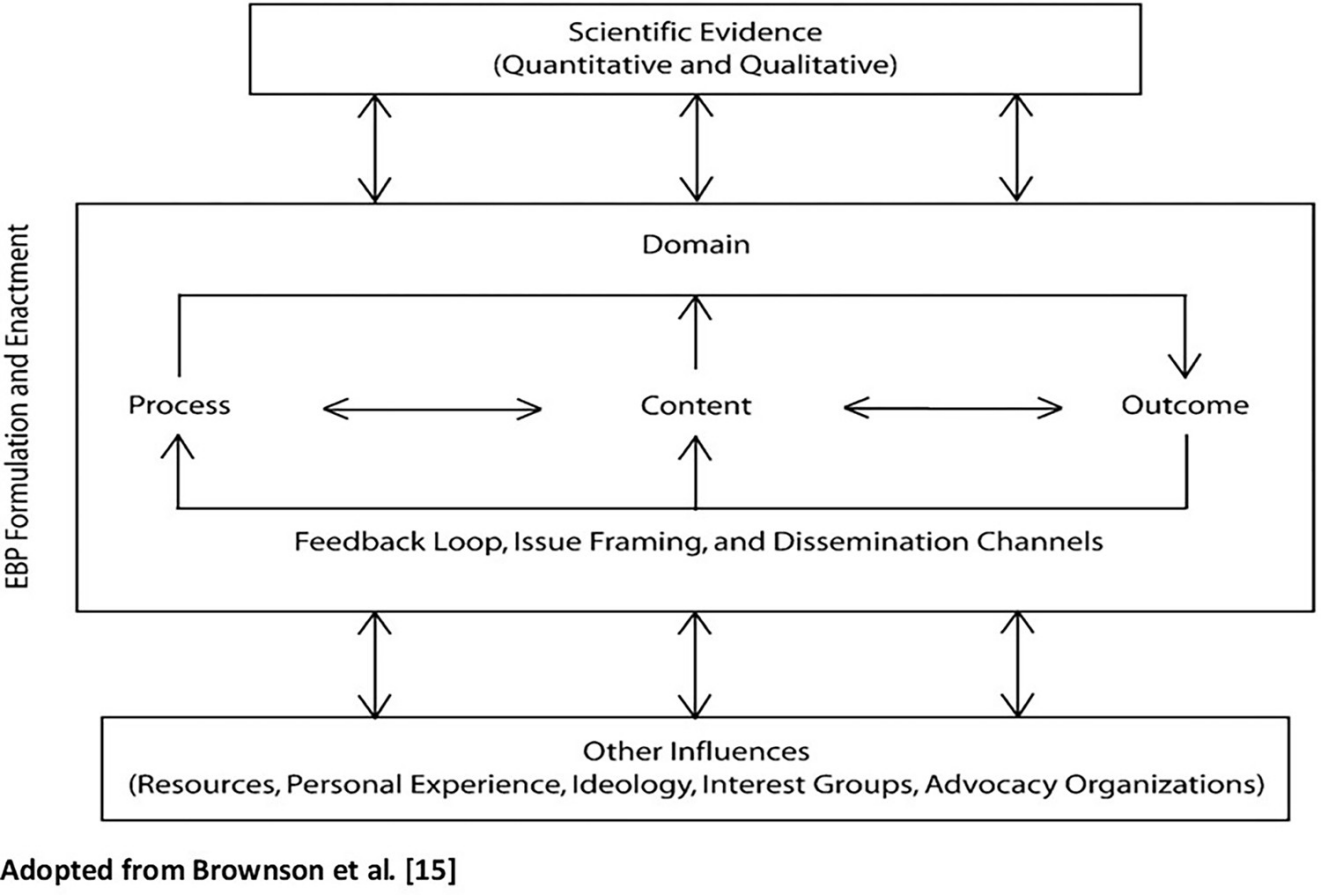


This paper adopts the domains of the evidence-based public health policy approach put forward by Brownson et al. [15]. See Fig 1 for details.

The authors state that evidence, which can be quantitative or qualitative, forms an integral aspect of all health policies. They conceptualize that the domains of evidence-based public health policy include policy process, policy content, and policy outcomes as a continuum. These three components are influenced by complex factors including setting the agenda, selecting a course of action from different alternatives, and political considerations such as ideologies, interests and personal experience through feedback loops and dissemination channels. The process domain involves documenting relevant political and other factors that influence the process as evidence for public health policy. The main objective of the process domain is to appreciate key issues which hinder effective policy adoption. For the process domain, the sources of evidence include key informant interviews, case studies, and surveys [15].

The policy content, the second domain of evidence-based public health policy in this framework, focuses on the use of data to make decisions on policy choices and interventions with the highest propensity to be effective. According to this conceptualization, examples of such sources of data include utilizing systematic reviews and content analysis to inform decision-making. This evidence will contribute to a better understanding of the evidence-based elements of existing or proposed policies [15].

The policy outcomes domain refers to the documentation of the outcomes or effects of policies already implemented for evidence purposes. Evaluating the impacts of implemented policies and interventions is critical to the evidence scenario as it can, for example, contribute to a better understanding of how behaviours have changed at individual and community levels over time. This will inform the adoption and implementation of future interventions in terms of which ones have been successful and which ones have not, leading to policy adaptation and modification. Brownson et al [15] suggest that data from surveillance systems as well as natural experiments monitoring policy-related endpoints are central to this domain. The framework approach is apt for this analysis because the interplay of the elements in the continuum could offer useful insights into the extent to which policies and programmes in Ghana have been underpinned by credible evidence.

## Methods

To gain deeper insights into how data are obtained for NCD policies in Ghana, a qualitative approach using key informant interviews and documents was adopted. The methods presented here is a brief description from a comprehensive study reported elsewhere [16].

### Key informant interviews

In Ghana, the Ministry of Health (MOH) is responsible for the development of policies and determination of health priorities while the Ghana Health Service (GHS) remains the statutory body responsible for implementation of policies. Ten (10) key informants were initially purposively sampled from MOH and GHS (5 each) based on this knowledge and experience related to NCD policy planning and implementation to cover the scope of NCD policy data in Ghana to reach theoretical saturation [17]. However, because initial key informants highlighted the role of health facility related data to informing the policy process, we recruited and interviewed two GHS health facility managers. A total of 12 interviews were therefore conducted with key informants from the MOH and GHS. Participants were selected based on their positions and roles in the management of NCDs at the national and subnational levels. Participants also needed to have been in their current role for at least two years to be selected for the interview. The profile of all study participants have been provided in (Table 1) below.

**Table 1:** Profile of the study participants.

Characteristics		N (%)
*Sex*	Male	10 (83)
	Female	2(17)
*Ethnic background*	Akan	6(50)
	Ewe	2(17)
	Ga	1 (8)
	Others	3 (25)
*Level of participants*	District	2(17)
	National	10(83)

Key informants were identified in collaboration with the management of MOH and GHS after formal letters had been submitted to seek permission and support for the study. Information sheets clearly outlining the nature of the research and the data to be collected was shared with management to help advise on suitable participants. Researchers then followed up on potential participants through face-to-face interaction, emails and phone calls where the purpose of the study was discussed with potential participants and questions answered. Interviews were scheduled with participants successfully recruited for the study on a date and time convenient for the participant at a location of their choice. All participants signed consent forms and provided consent for audio recording of the interview. All interviews were conducted by the first author.

### Documents

Documentary sources were used to support data from key informant interviews. Three categories of documents were used. The first was MOH and WHO country documents about NCD policies and programmes in Ghana. This category included policy documents such as the National NCD Policy, National NCD Strategy, alcohol, nutrition, and tobacco related policies, Standard Treatment Guidelines, and the WHO STEPs documents. The second set of documents came in the form of reports from the GHS detailing all the yearly NCD activities and progress in the management of information for NCD prevention. The third set of documents included Common Management Arrangement (CMA) documents, Programme of Work (PoW) documents, health financing, Regenerative Health and Nutrition Programme (RHNP) documents, and NCD Control Programme documents all of which provided insights into NCD policies and activities in Ghana.

### Data analysis

A qualitative content analysis approach geared towards the identification of relevant patterns and themes was used. Analysis was informed by a six-step adaptation of the framework method usually adopted in large-scale qualitative data [18]. This included initial preparation of data, importing interview transcripts and documents into NVivo 11 software, coding, developing a framework for analysis, sorting, and interpretation.

Initial data preparation involved transcription of all interviews into Microsoft Word document files by the first author. This also involved familiarization with the data as the audio recordings were played again to ensure they matched the transcripts, a process that allowed initial immersion in the data. For quality assurance purposes, the second and third authors also listened to audio recordings to ensure that transcripts were accurate. The first author imported transcripts and soft copies of documents into NVivo 11 Software (QSR International Pty Ltd). Data were coded (or labelled) when they were repeated in several transcripts or documents, intriguing or when respondents stressed their relevance. Running queries in the Software using frequently occurring words helped to identify initial patterns in the data. A framework for analysis based on putting codes together into higher-level categories was used, with the research purpose as a guide. The codes and categories were then organised using the Software into several nodes (containers in the Software for putting together related data to help identify useful patterns) as they connected to the study purpose. The sorting process involved dragging and dropping relevant paragraphs and sections into suitable nodes relating to the study purpose. Interpretation involved reviewing categories for key and interesting ideas which enabled parallels and differences to be drawn in the data. Although the first author spearheaded the whole analysis process (from data preparation to interpretation), periodic meetings were held with the team to engender consensus and accuracy.

### Ethics

Ethical approvals were obtained from the Ghana Health Service Ethics Review Committee (Ref No. GHS-ERC:15/06/17) and the University of Canterbury’s Human Ethics Committee (Ref No. HEC 2017/49).

### Findings

For confidentiality purposes, key informants (KI) have been anonymised to avoid identifiable information. Findings have been presented using the evidence-based public health policy domains framework [15]. In Ghana, however, different sources of data have been used to inform actions across the continuum, stressing the possibility for flexibility in the use of data in the policy process.

### Policy process

Surveys were found to be important sources of evidence for NCD policies in Ghana. The Ghana Demographic Health Survey (GDHS) “collects nationwide data on NCD risk factors–tobacco, alcohol, physical activity, fruits and vegetable consumption, and (for women only) body mass index as well as on blood pressure and biochemical measurements such as blood glucose, cholesterol and triglycerides” [8 p34]. A participant explained;

“*There are also surveys that are carried out*. *Let me say the GDHS which is carried out every 5 years…*..*that one we do not do it but it is a useful source of information on these conditions*. *The STEPS was very useful when we were developing the National NCD Policy and other programmes” [KI 8]*.

### Policy content

Although Good Practice Evidence for Interventions could be captured as part of case studies and under the process domain, they are usually adopted in the selection of interventions and policy content in Ghana. According to a participant [KI 6], best-practice evidence is equally important in guiding key decisions on NCDs in Ghana.

“*And then best practices are sources of information*. *For example*, *throughout the world*, *we know that vaccines are very cost-effective*. *So*, *if you have evidence like that*, *then it influences your policies*”.

Policy documents confirm the use of cost-effective vaccines as interventions. There are immunization programmes for hepatitis B which causes liver cancer, and efforts are being made to expand the coverage of immunization for human papillomavirus (HPV) vaccination for girls between the ages of 9 and 13 to prevent cervical cancer and throat cancer, with pneumococcal vaccine being introduced for children suffering from sickle cell disease [9 p 19].

We found that systematic reviews and meta-analyses form a crucial part of the evidence base for the selection of interventions in the policy process. For example, Ghana’s policy objectives for salt intake were informed by evidence from a 2003 systematic review by He and McGregor [9 p18]. Bosu’s review in 2010 is one of a number of reviews that informed the overarching NCD Policy of Ghana [9 p25].

### Policy outcomes

All 12 respondents (see Table 2) stated routine facility data as the most widely used source of data for NCD policies in Ghana, with key informants implying that this informs decisions across the policy continuum in Ghana. Documentary data shows that “routine morbidity and mortality data will be monitored… .… . with accurate, complete, and timely health information on morbidity and mortality of NCDs being routinely collected using the national health information system” [9 pp23-24]. One key informant noted;

“*We use routine health facility data and surveys as evidence for making policies and programs*. *Those ones are regular and periodic*, *but we aggregate them into yearly holistic data so that gives us some trends over the years*” [KI 3].

**Table 2:** Interview data.

Source/type of information	Frequency of responses	Percentage (%) of respondents
Routine health facility data	12	100
Surveys	8	67
Global burden of disease	5	42
Monitoring reports	3	25
Good practice evidence	1	8

A Centre for Health Information Management (CHIM) has been created under the supervision of the MOH for this [9]. A review of health information systems revealed the existence of “scattered information that did not communicate with each other, resulting in information redundancy, duplication and inconsistencies” [19 p68]. The District Health Information Management System (DHIMS), a comprehensive effort to develop software for generating reports on the activities of the municipal and district health authorities as well as health facilities at all levels of service delivery was, therefore, adopted. Data are ‘routinely collected within the context of the DHIMS and e-health’ (9 p24). A key informant added;

“*We have Health Information Officers who capture all the routine data directly at the facility level and report through the DHIMS” [KI 4]*.

The upgraded version, DHIMS-2, “allows data entry at multiple points in each district with a consolidation module at the regional and national levels” [20 p16]. The implementation of the DHIMS is, however, affected by human resource challenges [9]. Logistical constraints including inadequate computers in some areas have also been noted [19 p69]. Thus, the availability of routine data remains a challenge. A participant commented;

*“….It is difficult for us. There is this particular assignment WHO is helping the MOH to do, the Cross Programmatic Efficiency, how the Control Programmes (such as Malaria, TB, HIV/AIDS, and NCD Control Programmes) can work together to ensure efficiency because at the moment they are all working in parallel with their own plans, monitoring systems, supply chain systems….. These things are a waste of money and resources. So, WHO is helping us to use a model they have tried in one of the Asian countries and quite recently in South Africa. I tell you, getting data for this is hell” (KI 3)*.

Data quality issues were noted. A participant explained how this challenge is being handled;

“*We have validation teams at the facility and district levels who check questionable data*. *Also*, *within the DHIMS*, *there is an in-built validation system that streamlines the data*. *For example*, *when a disease that affects women is entered for men*, *the DHIMS will detect this error” (KI 6)*.

Despite these measures, it was found that the quality of routine data remains poor in Ghana. A participant (KI 7) explained;

“*We have problems with routine data quality in Ghana*. *Just by looking and comparing*, *you can detect common mistakes in the data provided*. *For example*, *you could find some indicators*, *if it is 2010*, *2011*, *2012*, *when you come to 2013*, *the 2012 figure that is brought forward changes*. *It means there is an issue with data quality”*.

The GHS is, however, working in collaboration with agencies to “develop a guideline for conducting data verification as well as coordinate the efforts of partners involved in data quality improvement activities to ensure standardization of procedures and outputs” [21 p114].

According to five respondents, the global burden of disease data from the US Institute of Health Metrics and Evaluation (IHME) underpinned the development of Ghana’s NCD policies. A participant (KI 1) commented as follows;

*“…… and even the Institute of Health Metrics and Evaluation (IHME), some of their projections also looked at Ghana having the NCD burden and current projections suggest that hypertension and diabetes burden are becoming larger and larger. So clearly, there was a need to do something, hence the National NCD policy”*.

Committees at the national level have been constituted to review studies to provide information on the burden of diseases in Ghana [22 p74]. To this end, “a draft report on burden of disease studies has been submitted after series of meetings, with capacity-building programmes in NCD control and field epidemiology already underway” [22 p74].

Monitoring reports (surveillance reports) of the MOH are also a source of data for NCD policies within the outcomes domain. A key informant [KI 9] explained;

“*Also*, *the rapid assessments*, *sometimes when we suspect something we go to the field and do some monitoring and those monitoring reports also help*. *These are done in the communities*”.

Documentary evidence supports this. In 2011, the “NCD Control Programme undertook monitoring visits to the Greater Accra Region in March, the Western Region in June, and the Volta Region in August” [23 p48].

## Discussion

Evidence shows that establishing national policies remains an important step in the prevention and control of NCDs [24]. However, the effectiveness of these policies in prevention efforts may be affected by the underpinning evidence. Policies that are framed on poor or inadequate evidence may be misguided, leading to poor outcomes. In many LMICs, the evidence base for NCD policies has been questioned [4]. The evidence-based public health approach offers a useful framework for understanding the types and role of evidence in the policy enterprise. Data sources for the process aspect of the evidence-based public health continuum include key informant interviews, case studies, and surveys of specific political contexts. Policymakers in Ghana admit that several consultations are made during the policy process with key informants in epidemiology and public health. Although research confirms this [25], we found that until now, such consultations are not documented for evidence purposes in Ghana. The failure to document consultations with key informants could affect the policy process in future since this has the potential to provide useful insights into the politics and prevailing atmosphere within which NCD policies are formulated. For example, policy experts believe that a failure to appreciate political factors impinging on the policy process accounted for poor health reform outcomes in many countries [26, 27]. Policymakers in Ghana have recognised this lacuna, and plan to document all the processes involved in the policy process for evidence purposes [25].

As part of the process domain, our findings show that data from national surveys are utilized in framing NCD policies in Ghana. This is important as experts have recommended the integration of NCD indicators into national surveys [28]. Several countries in Africa (including Malawi, Zambia, and Nigeria) have used data from the Global Youth Tobacco Surveys and the Global Adult Tobacco Surveys to guide their tobacco plans and other NCD policies [29]. However, research shows that little progress has been made in controlling diet, weight and physical activity, and that inadequate survey data on these risk factors is partially responsible for this [4]. Other studies have identified inadequate surveys as a contributory factor to the poor intersectoral action and implementation of NCD strategies in LMICs [30]. In Ghana, the STEPS Survey is conducted by the GHS under the aegis of the WHO but is tied to the availability of funds. Thus, more resources are needed to conduct these surveys as efforts need to go beyond the current one-off surveys for effective NCD policies. In contrast, South Africa is managing this situation well and has instituted a number of surveys (e.g. the South African Demographic and Health Survey, the Agincourt Health and Sociodemographic Survey and the South African Stress and Health Survey) to facilitate NCD plans and policies [31]. Considering that significant resources are required to undertake survey research in LMICs, policymakers could integrate the WHO STEPS into the more-established infectious disease systems in Africa as this offers a simple and standardized way of collecting and disseminating useful data on NCDs and risk factors [4].

Systematic reviews provide evidence about the content of health policies through assessment and analysis of research findings. In general, research shows limited utilization of systematic reviews in LMICs. In one study, it was found that 90% of NCD systematic reviews emanate from HICs, with just 0.15% undertaken in LMICs [32]. This is particularly concerning given that systematic reviews represent one of the strongest sources of evidence for health policies [33]. A possible reason for this could be the overemphasis on communicable, neonatal, and environmental disease research in many low-income countries, with NCD research receiving attention only recently [5–7]. The need to have a systematic and coherent approach to the utilization and integration of evidence from systematic reviews is, therefore, paramount. Unfortunately, except for South Africa which has embarked on a concerted effort to integrate systematic reviews into the policy process through its Initiative for Systematic Reviews and Rapid Evidence Synthesis [34], many African countries have no systems for this purpose. Investments in cutting-edge research and policy leadership are required to make this happen.

Within the outcomes domain, the present study found routine health facility data detailing the number of people who suffer or die from NCDs each year as the most widely used source of data for NCD policies in Ghana. Key informants in Ghana use this across the continuum as it influences discussions with stakeholders and provides insights into policy outcomes. The challenge in Ghana and other LMICs, however, is that many deaths occur at home, and these go unrecorded due to poor vital registration systems [35], contributing to the poor availability and accuracy of data for NCD policies. The poor availability of cause-specific mortality data has been noted particularly in Africa and Asia [36]. This has affected global cause-specific estimates as data from these continents remain unreliable, resulting in great uncertainty [37]. Consequently, verbal autopsies, where relatives, caregivers, and other close witnesses of the dead are interviewed for cause-of-death information, have been suggested [38]. This is important because it is possible to access data on relevant death trends by examining verbal autopsy reports from locations that collect such information routinely at the population level through longitudinal analysis, and this prevents the challenge of having to impose an overall mortality envelope associated with modelled estimates [39].

The Global Burden of Disease (GBD) estimates represent a useful and realistic source for NCD data because it reflects disability as well as mortality. It provides an independent, evidence-based approach to public health policy and planning, with some advocates believing that this is a more realistic estimate of disease than the disease-specific information that is usually published [40]. In Ghana’s case, the GBD estimates became a useful source of data on the leading causes of death in a time of health transition. Like Ghana, South Africa used GBD estimates to guide NCD activities. For example, through the 2000 Burden of Disease Study, a rigorous review of cause-of-death estimates in South Africa adjusted for under-registration and misclassification of causes were combined with available morbidity data for extrapolation of estimates of Disability Adjusted Life Years (DALYs) and was used as evidence for the development of NCD policies [31]. It may be beneficial for LMICs to pay attention to the in-country (local) burden of disease analysis since this can help in contextualizing and making meaning of the global estimates, contribute to setting appropriate NCD priorities and determine the impacts of implemented policies on specific population segments. However, in many African countries, political factors have contributed to the use of global estimates in policy development and implementation rather than local data. For example, Juma et al [28] identified high political involvement in NCD policy development in Africa. However, in line with our findings, they reported that poor local data and capacity affect policy implementation. Limited political commitment, inadequate resources and industry interference have all been implicated in poor implementation of NCD policies by these authors. There is the need, therefore, to commit resources to develop local capacity for generating robust local data to support policy development, planning and implementation.

Policymakers have been encouraged to adopt ‘natural experiments tracking policy-related endpoints’ in the policy process [15 p1578]. Yet key informants and documentary sources revealed little application of this in the policy process of Ghana. In general, the use of natural experiments resonates with the implementation science technique which represents the application of some of the principles of evidence-based medicine to health. It involves utilizing randomized controlled trials in community settings to evaluate the effectiveness of interventions and to ensure fidelity (implementation has occurred as planned). Often LMICs have used traditional models including the top-down, bottom-up and integrated systems of implementation in the policy process [25]. Thus, implementation science represents a paradigm shift from the pragmatic, even hopeful, approaches adopted in traditional programming. Our findings here correspond to findings elsewhere as implementation science techniques have been utilized in NCD prevention and control efforts mainly in advanced countries. For example, implementation science approaches and models underpin the American Diabetes Association Standards of Care and Society of Hospital Medicine Best Practices [41]. Empirical studies have yielded promising results from the application of implementation science approaches in the NCD prevention in high-income settings, with Magee et al [42] providing evidence of a successful framework for diabetes education in the US. There is, therefore, more potential for Ghana and other LMICs to utilize implementation science frameworks in the policy process especially when one considers the fact that a key challenge in LMICs is the implementation of NCD policies [4, 13, 25, 28].

Quality issues were noted in NCD data in Ghana. In general, data for public health policies in LMICs have failed to meet WHO criteria for data quality [43], with inaccuracies and incompleteness among the issues of concern. In the present study, this was influenced by human resource and logistical challenges which are consistent with findings elsewhere [25, 28]. However, other reasons have been noted in this and include, for routine mortality data, the selection of a single cause of death. In one study in South Africa, it was found that of 38 people recorded as being HIV positive, 6 died from causes not directly related to HIV but stroke, hypertension, and renal failure [44]. The implication here is that care must be taken in assigning codes in the case of the elderly who usually suffer from multiple diseases. Jha [39] attributes the problem to historical neglect of NCDs and a lack of resources in LMICs. Whatever the reasons, the implications on NCD policies are dire, limiting the use of data for planning, monitoring, and evaluation purposes. To improve the quality of cause-specific mortality information, a concerted effort must be made to widen coverage by increasing the percentage of mortality certified by physicians, educating personnel in the data capturing process on the relevance of accurate reporting, and eliminating ill-defined codes. Ghana could learn from LMICs such as Rwanda which has implemented a comprehensive programme integrating cause-specific mortalities into programmes while simultaneously strengthening the health system to address other conditions [45].

Within the context of the delivery of health services to people with NCDs in Ghana and other LMICs, a concerted effort must be made to invest in the evidence scenario through training of appropriate personnel and logistics. This will not only contribute to the provision of credible evidence for policy development, planning and implementation of health interventions but will also help provide effective and timely public health response to support people with NCDS before their conditions escalate to uncontrollable levels. This is crucial as failure to respond appropriately to rising NCDs through evidence-based policies, programmes and services could lead to catastrophic health expenditure and other economic and social costs on already impoverished families and communities. We believe the present study provides relevant insights into the current state of NCD policies and their underlying evidence. For example, the current overreliance on occasional demographic surveys has proven not to be enough to support the planning and implementation of services to combat NCD risk factors. There is, therefore, the need to invest more in national surveys to help address the ever-mounting incidence of risk factors since programmes targeting lifestyle-related risk factors through policies have been known to be effective in combating NCDs in populations and individuals if built on credible evidence [46]. Our study does not only provide insights into how existing sources of data could be strengthened for policy purposes but also throws light on new approaches of evidence that could support the design and implementation of NCD health services, and which are yet to be considered in NCD management in Ghana and Africa. For example, policymakers should consider the benefits of utilizing implementation science techniques in developing interventions especially in community settings compared to traditional implementation approaches. We believe exploring other approaches of evidence synthesis and utilization could go a long way to impact positively on the effectiveness of policies and interventions for chronic disease management in LMICs.

### Limitations and future research considerations

There is a possible limitation in this study that could be addressed in future research. Apart from the MOH and the GHS, there are other agencies playing crucial roles in NCD management and prevention in Ghana. Our study mainly focused on mainstream governmental institutions saddled with the responsibilities of policy development and implementation for the health sector. In particular, the Christian Health Association of Ghana (CHAG), a key implementation agency, is an umbrella body comprising faith-based organizations that provide health care services to Ghanaians. It would have been beneficial to interview representatives from these institutions to illuminate the findings of the study. However, covering all policy development and implementation agencies require more resources and time, hence beyond the scope of this study. We believe that exploring the role of faith-based organizations and private stakeholders in policy development and implementation constitute a gap to be explored by future researchers in Ghana.

## Conclusion

With limited research into the use of evidence in NCD policies, our study aimed to explore how this process takes place in the Ghanaian health system. Guided by Brownson et al [15], our analysis reveals surveys, routine health facility data, systematic reviews, surveillance reports and burden of disease estimates as the main sources of data for NCD policies in Ghana. The policy process is, however, affected by a limited emphasis on case studies, content analysis, and the application of implementation science techniques. Although the signs are encouraging as relevant institutions and systems such as the CHIM and DHIMS have been established, the availability and quality of data need improving. Considering that NCDs are a relatively new cluster on the disease roster in Ghana and many LMICs, more data are needed for policies and programs. It would be necessary to not only strengthen existing systems and institutions by providing the requisite human resources and logistics but also widen the scope to include, among other sources, verbal autopsies and natural experiments tracking policy-related endpoints through implementation science approaches. This becomes even more important since policies and programs forged upon incomplete and poor-quality data could jeopardise implementation efforts and erode gains made. More investments are, however, needed at the national and district levels for this to be achieved.
